# Physician perspectives on de-intensifying diabetes medications

**DOI:** 10.1097/MD.0000000000005388

**Published:** 2016-11-18

**Authors:** Natalia Genere, Robert M. Sargis, Christopher M. Masi, Aviva G. Nathan, Michael T. Quinn, Elbert S. Huang, Neda Laiteerapong

**Affiliations:** a Section of General Internal Medicine; b Section of Endocrinology, Diabetes, and Metabolism, Department of Medicine, University of Chicago, Chicago; c NorthShore University HealthSystem, Evanston, Illinois.

**Keywords:** diabetes, endocrinology, outcomes research, patient centered care, patient preference, patient satisfaction, quality of care

## Abstract

Guidelines for diabetes care recommend that physicians select individualized glycemic goals based on life expectancy, diabetes duration, comorbidity, and resources/support. When patients have stable hemoglobin A_1C_ (HbA_1C_) levels, guidelines lack recommendations on when diabetes medications should be de-intensified.

To understand physicians’ perspectives on de-intensifying diabetes medications in patients with type 2 diabetes.

Cross-sectional survey, (February–June, 2015).

Academic medical center and suburban integrated health system.

Primary care and endocrinology physicians.

Physicians’ self-reported: awareness, agreement, and frequency of individualizing HbA_1C_ goals; practice of de-intensifying diabetes medications; HbA_1C_ values at which physicians de-intensify diabetes medications; and other patient factors physicians consider when de-intensifying diabetes medications.

Response rate was 73% (156/213). Most physicians (78%) responded they were familiar with recommendations to individualize HbA_1C_ goals. For patients with stable HbA_1C_ levels, 80% of physicians reported they had initiated conversations about stopping medications; however, physicians differed in predefined HbA_1C_ levels used to initiate conversations (HbA_1C_ < 5.7%: 14%; HbA_1C_ < 6.0%: 31%; HbA_1C_ < 6.5%: 22%; individualized level: 21%). In multiple logistic regression, women physicians (odds ratio [OR] 3.0; confidence interval [CI] 1.1–8.2; *P* = 0.03) and physicians practicing fewer than 20 years (OR 2.8; CI 1.01–7.7; *P* = 0.048) were more likely to report de-intensifying diabetes medications.

Individualizing glycemic goals and de-intensifying treatments are concepts well accepted by physicians in our sample. However, physicians vary considerably in reporting how they carry out recommendations to individualize and may be missing opportunities to stop or taper diabetes medications based on patients’ individualized glycemic goals.

## Abstract 2

Supplemental Digital Content is available in the text

## 1 Introduction

Diabetes affects 29.1 million adults in the United States and costs $245 billion annually.^[1]^ Nearly 30% of these costs are attributable to diabetes medications and supplies.^[1]^ The central role of diabetes medications is to control glycemic levels in order to prevent downstream diabetic complications. However, in the last decade, major trials have provided conflicting evidence regarding the clinical effects of intensive glycemic control.^[2–6]^ These trials have found that the microvascular, cardiovascular, and mortality benefits of intensive control require up to 10 or 20 years to emerge and that benefits of intensive control may only exist for patients with newly diagnosed diabetes.^[2,7,8]^ More recent trials conducted in older patients with high levels cardiovascular risk found that intensive glycemic control may, at best, decrease cardiovascular events and improve surrogate microvascular endpoints, like microalbuminuria, and, at worst, lead to increased mortality.^[3–6]^

Due to the conflicting clinical trial evidence, in 2012, a position statement was released by the American Diabetes Association (ADA) and European Association for the Study of Diabetes that emphasized the importance of individualizing glycemic management for type 2 diabetes based on specific patient characteristics (e.g., age, comorbid conditions, life expectancy, micro- and macro-vascular complications, resources, and support).^[9]^ This position statement was adapted into ADA diabetes care guidelines^[10,11]^ which are consistent with those previously published by the American Geriatrics Society (AGS)^[12]^ and Veteran Affairs/Department of Defense.^[13]^

In clinical practice, many patients may have initiated and intensified diabetes treatment prior to current recommendations to individualize glycemic control. As a result, many patients may have surpassed their individualized glycemic goal and continue to take intensive regimens, which has been defined as diabetes overtreatment. Our previous research suggests that nearly one-quarter of older US adults with diabetes may be exposed to diabetes overtreatment.^[14]^ Diabetes overtreatment increases the risk for hypoglycemia, which is now a leading diabetes complication,^[15–19]^ requires extensive lifestyle modifications,^[20]^ and places significant financial burden on patients.^[21]^

The high prevalence of the overtreatment scenario raises important clinical questions regarding when and how clinicians should de-intensify therapy. Few guidelines provide recommendations on how to de-intensify or address hypoglycemia risk.^[22]^ The most relevant recommendations are from the AGS, which cautions against polypharmacy,^[12]^ and Choosing Wisely/AGS, which recommends against using medications other than metformin to achieve HbA_1C_ < 7.5% in most older patients.^[23]^

Because of the importance of, and limited guidance on, de-intensifying medications in patients with type 2 diabetes, we surveyed primary care and endocrinology physicians regarding their perspectives and practices of de-intensifying diabetes medications.

## 2 Methods

### 2.1 Study design and participants

This was a cross-sectional survey of primary care physicians and adult endocrinologists at an urban academic medical center (University of Chicago Medical Center [UCMC]) and a suburban integrated health system (NorthShore University HealthSystem [NSUHS]). The survey was administered in three waves between February 2015 and June 2015. At UCMC, primary care physicians included internal medicine physicians who practiced at a single hospital-based clinic; endocrinologists at UCMC also practiced at a single hospital-based clinic. Primary care physicians at NSUHS included internal medicine, geriatrics, and family medicine physicians. At NSUHS, there were 27 primary care clinic practices which averaged 5.4 providers per clinic (range, 1–9) and 6 endocrinology clinic practices which averaged 2.3 providers per clinic (range, 1–3). The study was approved by the University of Chicago and NorthShore University HealthSystem Institutional Review Boards.

### 2.2 Survey development

Survey questions were developed by a panel of internal medicine physicians and experts in health sciences research and diabetes affiliated with the National Institutes of Health-funded Chicago Center for Diabetes Translation Research (P30 DK092949). Cognitive testing was then performed using the “think aloud” method with practicing primary care physicians.^[24]^ Survey questions were iteratively revised after each cognitive interview until all survey questions reflected the content intended by investigators.

### 2.3 Survey content

Main outcomes were physician self-reported awareness of, agreement with, and frequency of individualizing HbA_1C_ goals; practice of de-intensifying diabetes medications; HbA_1C_ values at which they de-intensify diabetes medications; and other patient factors physicians consider when de-intensifying diabetes medications. The survey included the definition of individualizing HbA_1C_ as “choosing a HbA_1C_ goal for each patient based on their characteristics.” Physicians were asked about their familiarity with individualizing HbA_1C_ goals (“yes”/“no”), whether they agreed with the concept of individualizing HbA_1C_ goals for each patient based on their characteristics. (Five-point Likert scale, “strongly disagree” to “strongly agree”), and how frequently they individualized HbA_1C_ goals (5-point Likert scale, “rarely” to “always”) (eSupplement 1).

After being asked about individualizing HbA_1C_ goals, physicians were asked about their perspectives regarding diabetes medication de-intensification. Specifically, physicians were asked, “In general, if your patient with type 2 diabetes has a stable A_1C_ level for 1 year, do you ever initiate conversations about discontinuing or reducing the dose of their diabetes medications?” (“Yes”/“no”). For physicians who responded “yes”, they were asked at what HbA_1C_ this conversation was initiated (“<5.7%,” “<6.0%,” “<6.5%,” “<7.0%,” “<8.0%,” “the HbA_1C_ level depends on the patient's characteristics,” or “other”). If physicians selected an HbA_1C_ value we considered this value “predefined,” as in defined *a priori* without patient characteristics taken into account. Also, physicians were asked to indicate in what other situations they initiated conversations about de-intensifying diabetes medications; answer options included when patients had potential side-effects from medications, risk for polypharmacy, medication non-adherence, reduced life expectancy, concerns about medication costs, or other.

Physicians also reported their sex, specialty, years in practice, as well as patient panel characteristics (total panel size and percent patients with age 65 years or older).

### 2.4 Survey recruitment and administration

At UCMC, paper surveys were distributed in-person and by inter-office mail. At NSUHS, paper surveys were distributed to physician clinic leaders at a regularly scheduled monthly meeting. The lead physicians then distributed surveys to their clinic partners. At both sites, physicians were reminded via email three times to return surveys and a second paper survey was mailed with the third reminder email to non-respondents. A $10 financial incentive was included in the first survey. Return of the survey was presumed to be informed consent.

### 2.5 Statistical analysis

Summary statistics were calculated using means and proportions as appropriate. Bivariate and multivariate relationships between physician/practice characteristics and awareness, agreement, and frequency of individualizing HbA_1C_ goals; practice of de-intensifying diabetes medications; and HbA_1C_ values at which they de-intensify diabetes medications were calculated using chi-square analysis or Fisher exact tests, and multiple logistic regression. In bivariate and multivariate analysis, physician and practice covariates were dichotomized at the median, such that years in practice was defined as <20 versus ≥20 years, panel size was defined as ≤1000 versus >1000 patients, and percent patients age 65 years or older was defined as ≤40% versus >40%. Level of agreement with and frequency of individualizing goals were also dichotomized (somewhat/strongly agree vs. not and most of the time/always vs. not). Practice site and specialty covariates were defined as academic medical center versus suburban integrated health system and endocrinology versus primary care, respectively. *P* values less than 0.05 were considered statistically significant. Analysis was performed using SAS 9.4 (Cary, NC).

## 3 Results

**Figure 1:**
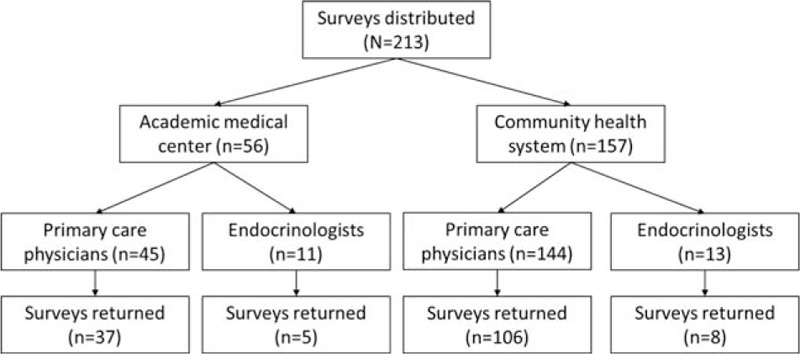
Physician survey respondent flow chart.

The overall response rate was 73% (overall: 156/213; primary care physicians: 143/189, and endocrinologists: 13/24). Response rates did not vary by study site (NSUHS 73% and UCMC 75%) but did vary by specialty (primary care physicians vs. endocrinologists: 76% vs. 54%; *P* = 0.03) (Fig. 1).

### 3.1 Physician and practice characteristics

Among survey respondents, 92% were primary care physicians and 8% were endocrinologists (Table 1). About two-thirds of respondents were internists (67%), 21% were family medicine physicians, 1% were geriatricians, and 3% reported more than 1 primary care specialty. About half of physicians were female (53%). Physicians varied widely in their years in practice with 27% practicing for fewer than 10 years, 26% for 10 to 20 years, 16% for 20 to 24 years, and 31% for 25 years or longer. About one-quarter of physicians reported caring for fewer than 500 patients and 40% of physicians reported caring for over 1500 patients. About one-third of physicians each reported 21% to 40% or 41% to 60% of their patients being aged 65 years or older.

**Table 1:**
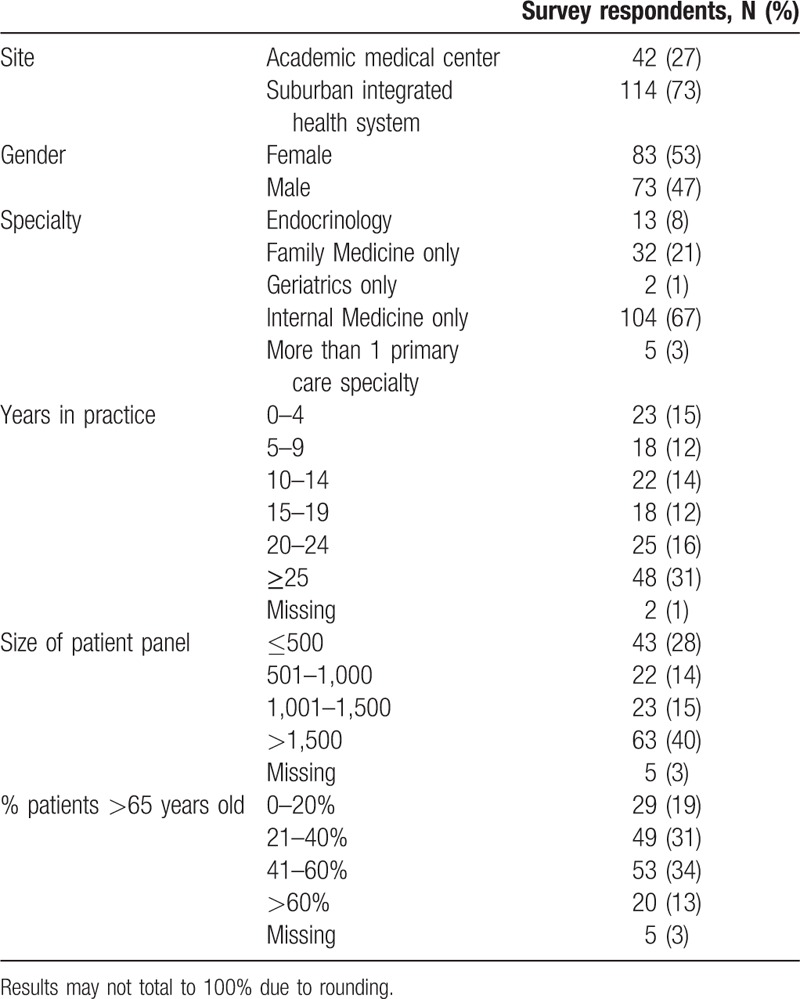
Primary care and endocrinology physician and practice characteristics (N = 156).

Compared with NSUHS, UCMC physicians were in practice for fewer years (<20 years: UCMC vs. NSUHS, 66% vs. 48%; *P* = 0.047), had smaller patient panels (≤1000 patients: 81% vs. 28%; *P* < 0.001), and had larger proportions of older patients (>40% of practice is >65 years old: 62% vs. 43%; *P* = 0.04). UCMC and NSUHS physicians did not differ by sex.

### 3.2 Individualizing HbA_1C_ goals

Most physicians (78%) responded that they were familiar with the concept of individualizing HbA_1C_ goals (Table 2). Among those familiar with HbA_1C_ individualization, 82% agreed with the concept, whereas only 13% disagreed with it. Physicians familiar with individualizing HbA_1C_ goals reported using individualized goals “most of the time” (48%) or “always” (21%).

**Table 2:**
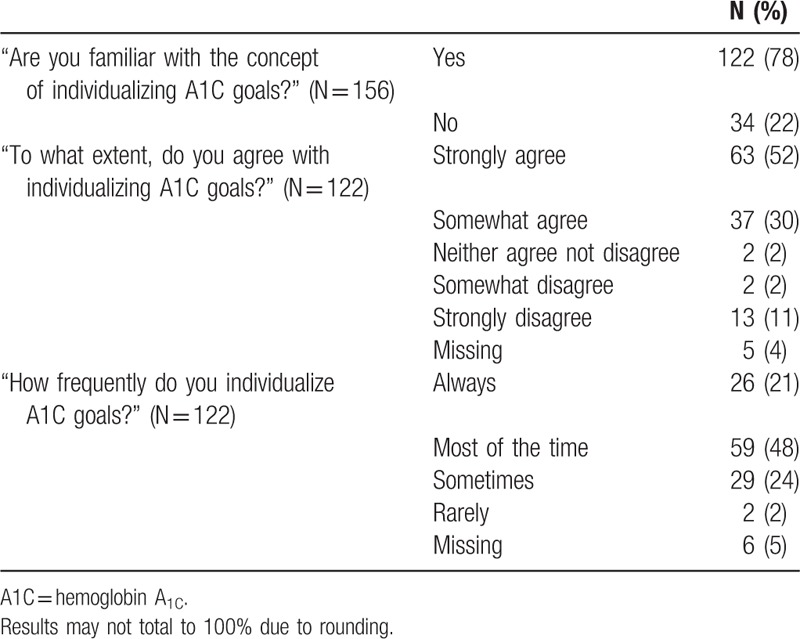
Physicians’ perspectives and practices of individualizing HbA_1C_ goals.

In bivariate analysis, familiarity with individualizing HbA_1C_ goals was associated with practice site, patient panel size, and percentage of patients aged 65 years or older. However, in multivariate analysis, only practice site was associated with familiarity with individualizing HbA_1C_ goals (academic medical center vs. suburban integrated health system: odds ratio [OR] 12.6, 95% confidence interval [CI] 1.5–103.8; *P* = 0.02).

Agreement with individualizing HbA_1C_ goals was not associated with physician or practice characteristics in bivariate analysis. However, fewer years in practice was associated with agreement with individualizing HbA_1C_ goals in multivariate analysis (≤20 vs. >20 years, OR 3.4; CI 1.01–11.4; *P* = 0.049).

Frequency of individualizing HbA_1C_ goals was associated with specialty type, years in practice, and percentage patients aged 65 years or older in bivariate analysis. However, these relationships were not significant in multivariate analysis.

### 3.3 De-intensifying diabetes medications

The majority of physicians (80%) reported that they initiated conversations about discontinuing or reducing the dose of diabetes medications for patients with stable HbA_1C_ values (Table 3). The majority of physicians (74%) reported initiating conversations about medication de-intensification based on predefined HbA_1C_ values; only one-fifth of physicians (21%) reported initiating this conversation based on individualized HbA_1C_ levels. Physicians used a wide range of predefined HbA_1C_ levels to initiate conversations on de-intensifying medications; the most frequently used HbA_1C_ levels were <6.0% (31%), <6.5% (22%), and <5.7% (14%). Other HbA_1C_ values were rarely used (<7.0% [5%]; <8.0% [2%]). Physicians also initiated conversations about de-intensifying medications when patients had possible medication side effects (90%), limited life expectancy (73%), polypharmacy (62%), concerns about medication costs (56%), or medication non-adherence (52%).

**Table 3:**
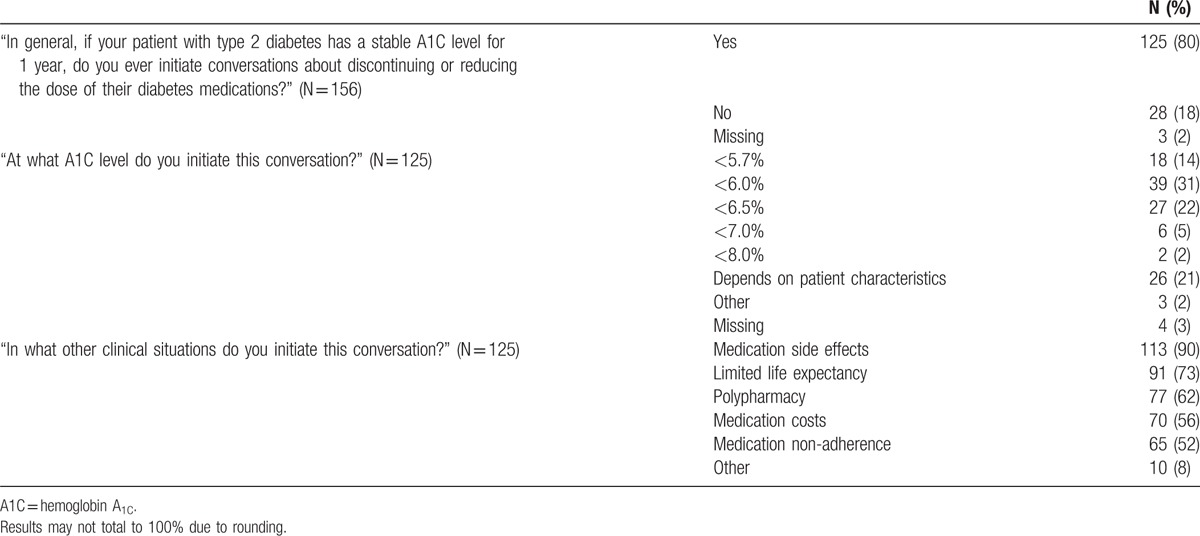
Physicians’ perspectives and practices of de-intensifying diabetes medications.

In bivariate analysis, initiating a conversation about medication de-intensification was associated with physician sex and years in practice. These relationships remained significant in multivariate analysis, such that women physicians (vs. men) and physicians practicing for 20 years or less (vs. >20 years) were more likely to initiate conversations about de-intensifying diabetes medications (OR 3.0, CI 1.1–8.2; *P* = 0.03, and OR 2.8, CI 1.01–7.7; *P* = 0.048, respectively). Also, practicing at an academic medical center versus suburban integrated health system was significantly associated with conversations about medication de-intensification (OR 4.4, CI 1.1–17.9, *P* = 0.04).

## 4 Discussion

Our study suggests that most physicians in our sample are aware of guidelines advocating individualized HbA_1C_ goals in patients with type 2 diabetes and are considering medication de-intensification in patients with stable HbA_1C_ levels. Many physicians in our sample used predefined HbA_1C_ thresholds to trigger discussions about diabetes medication de-intensification, even though they were also aware of patient characteristics important for individualizing diabetes care. Inconsistent report of de-intensification practices in our sample may reflect variable physician awareness of existing guideline recommendations as well as a genuine clinical uncertainty over when and how to de-intensify diabetes medications.

Although clinical guidelines supporting individualization are reported to be widely used by physicians within our sample, over 20% of physicians still reported being unaware of the concept of individualizing glycemic goals. Physicians that do not individualize goals may pursue overly aggressive treatments with uncertain benefits and potential harm.^[5,15,17]^ We also found that physicians considered several different predefined HbA_1C_ thresholds when deciding to initiate conversations about medication de-intensification. Most physicians chose very reasonable HbA_1C_ values, for example, <6.0% or <6.5%; however, these predefined values may leave patients at risk for overtreatment, compared with using an individualized approach. For example, an older adult with a history of heart disease and an individualized HbA_1C_ goal of <8.0% may be overtreated if their physician does not consider de-intensifying medications until their HbA_1C_ level is 6.4%. Our previous study showed that applications of leading guidelines would lead to less intensive glycemic targets (e.g., HbA_1C_ <8% instead of <7%) for up to 70% of treated US adults with diabetes.^[14]^

Several physician and practice characteristics were associated with familiarity and agreement with individualization, and initiating conversations about de-intensifying medications. We found that female physicians were more likely to report initiating conversations about de-intensifying medications. Few studies have examined sex differences in the practice of medicine; however, extant literature suggests that women physicians may conduct longer visits and ask more questions than men physicians,^[25]^ and are more likely to discuss physical activity and lifestyle modification.^[26–28]^ We also found that physicians practicing for fewer than 20 years were more likely to de-intensify medications, which may be related to physicians’ anchoring based on the state of diabetes care guidelines and evidence during their training period. Our finding that academic medical center physicians were more aware of the concept of individualization and de-intensification may be specific to our physician sample. Several clinical researchers in the study are translational diabetes experts who have influenced the practice of other clinicians. Further research examining actual physician practices is necessary to elucidate the generalizability of our findings.

One important question raised by this study is whether or not reports of de-intensification are consistent with actual clinical practice. Physicians may often worry about the balance between meeting performance metrics for the practice with the need for glycemic individualization for patients.^[29]^ In prior research conducted at the academic medical center, physicians reported that they individualized glycemic and blood pressure goals by health status among older patients, but a chart abstraction study revealed that the proportion of patients achieving HbA_1C_ <7.0% was nearly identical across health status categories such as physician-estimated life expectancy, age, level of comorbidity, and functional status (∼35%).^[30]^ More recent national studies suggest that this pattern of diabetes care persists for older adults. In the National Health and Nutrition Examination Survey from 2001 to 2010, the proportion of older patients achieving an HbA_1C_ <7.0% was found to be 61% overall; this was identical across health status groups and did not change over time.^[15]^ Of the patients with HbA_1C_ <7.0%, the rate of insulin or sulfonylurea use was 55% overall and across health status groups. Studies from the Department of Veteran Affairs have also revealed a high prevalence of potential glycemic overtreatment in adults with diabetes.^[17,31]^

Possible reasons for glycemic overtreatment include physicians’ unawareness of appropriate individualized targets, patients’ preferences which lead to overly aggressive targets, and clinical inertia. Physicians that are aware of guidelines to individualize diabetes care may still need decision support to facilitate the selection of individualized glycemic goals and formalized protocols to de-intensify treatments.^[32]^ Initiating conversations about diabetes medication de-intensification are also limited by time demands of clinical practice, as they require a discussion of risks and benefits of de-intensification as well as deciding which medication to eliminate.

Currently, there is no established framework for how to approach medication de-intensification in patients with type 2 diabetes. We propose an algorithm to guide providers in de-intensification of diabetes medications; our goal is to provide an approach that guides clinical decision-making and is not meant to be absolute. We suggest that clinicians and patients first agree on an individualized HbA_1C_ goal that takes into account the patient's age, comorbid conditions, life expectancy, micro- and macro-vascular complications, resources, and support. Physicians should then calculate each patient's “delta HbA_1C_,” which is the difference between the patient's individualized HbA_1C_ goal and their measured HbA_1C_. If this “delta HbA_1C_” is greater than the average HbA_1C_ lowering of one of their diabetes medications, a 3-month trial of discontinuing the medication may be appropriate. On average, most diabetes medications lower HbA_1C_ by about 1% with a range from 0.8% to 1.5% depending on medication class.^[33]^ Physicians should also consider medication side effect profiles when deciding which medication to discontinue. This recommendation should be framed as a “trial” that may succeed if paired with healthy lifestyle practices. It should also be emphasized that the de-intensification trial is not evidence that their diabetes is cured, since remission rates for diabetes are low,^[34,35]^ but that the risks of the given medication, such as weight gain, diarrhea, and hypoglycemia, may outweigh its potential benefits. Patients should also be counseled to monitor their blood glucose levels after a medication has been discontinued and to complete a follow-up HbA_1C_ test in 3 months. Future research should examine the feasibility and success of our proposed framework in clinical practice.

Because of the clinical importance of de-intensifying diabetes medications safely and in the appropriate patients,^[15–19]^ it is essential that best practices for de-intensification be established. The questions of when to discontinue, which medication to eliminate, and how to monitor patients’ progress would be best addressed with a randomized clinical trial. Once best practices are established, the development of clinical decision support tools and physician education will be crucial in translation of data into improving clinical practice. Furthermore, because education alone is unlikely to improve quality of care,^[36]^ mechanisms of feedback to physicians should also be used to ensure practice change. Since quality and performance metrics are entrenched in physician practice, part of changing practice must include public policy efforts to educate the developers of these metrics.

This study has several strengths and limitations. This physician survey was conducted in 2 different clinical sites among physicians with different panel sizes, patient ages, and years in practice. The response rate was very good for a physician survey overall. However, there were fewer endocrinologists and geriatricians in our sample; larger sample sizes of endocrinologists and geriatricians would be beneficial in future studies to determine if attitudes, beliefs, and practices differ by specialty. Also, the academic medical center physicians were likely biased in their awareness of the concept of individualization and de-intensification due to the local emphasis on diabetes research. Because of the nature of the study, a cross-sectional survey of physicians, physicians’ report of knowledge, attitudes, and behaviors may not translate into actual clinical practice. As with all self-report surveys, responses are subject to social desirability bias, such that the awareness, agreement, and frequency of individualizing HbA_1C_ goals and practice of de-intensifying medications may be over-estimated. Also, for physicians who were unfamiliar with individualization, we did not ascertain their willingness to learn about the concept; this will be an important follow-up question in future studies, as it may clarify the extent to which physicians are open to changing their practices. Finally, while this survey provides preliminary information about physician perspectives on de-intensifying diabetes medications, many more questions exist about the safety and efficacy of such practices. Further studies should examine which populations may safely discontinue diabetes medications without adverse hyperglycemic events.

In summary, de-intensifying diabetes medications is an important concept that has only recently received attention. We found that primary care physicians and endocrinologists frequently individualized HbA_1C_ goals; however, in general, decisions to de-intensify diabetes medications were driven by predefined HbA_1C_ thresholds, rather than by individualized goals. We propose that the difference in individualized HbA_1C_ goals and measured HbA_1C_ values should guide the de-intensification of diabetes medications. Further research is needed to assess the utility of this approach in clinical practice.

## Acknowledgments

NG and NL had full access to all of the data in the study and take full responsibility for the integrity of the data and accuracy of the data analysis. NL, RMS, CMM, AGN, ESH, MQ made substantial contributions to the conception and design. NG, NL made substantial contributions to the analysis and interpretation of data. NG drafted the manuscript. NL, RMS, CMM, AN, ESH, MQ provided critical revision of the manuscript for important intellectual content. AN collected data and provided administrative support. NL obtained funding. RMS does participate in the “National Pharmacy and Therapeutics Committee” for CVS Health, which is an independent advisory group. No other persons have contributed substantially to this work. Some contents of this work was presented in May 2016 as a poster in the Society of General Internal Medicine National Conference.

## Supplementary Material

Supplemental Digital Content
