# Supplementary material for: Physician perspectives on de-intensifying diabetes medications
Source: Medicine (Baltimore). 2016 Nov 18;95(46):e5388. doi: 10.1097/MD.0000000000005388 (PMC5120930; doi:10.1097/MD.0000000000005388)
Supplement: Supplemental Digital Content [file medi-95-e5388-s001.docx]

**eSupplement 1. Survey Questions**

1. What is your specialty? (Check all that apply)

- Endocrinology
- Family Medicine
- Geriatrics
- Internal Medicine
- Pediatrics

1. How many patients in total do you care for (estimated panel size)? (Check one)

| - 0-100 | - 101-250 | - 251-500 | - 501-1000 | - 1001-1500 | - >1500 |
| --- | --- | --- | --- | --- | --- |

1. What percent of your patients is >18 years of age? (Check one)

| - 0-20% | - 21-40% | - 41-60% | - 61-80% | - 81-100% |
| --- | --- | --- | --- | --- |

1. What percent of your patients is >65 years of age? (Check one)

| - 0-20% | - 21-40% | - 41-60% | - 61-80% | - 81-100% |
| --- | --- | --- | --- | --- |

1. What percent of your patients has Type 2 diabetes? (Check one)

| - 0-20% | - 21-40% | - 41-60% | - 61-80% | - 81-100% |
| --- | --- | --- | --- | --- |

1. What percent of your patients has both Type 2 diabetes and diabetic complications (e.g., eye disease, heart disease, heart failure, kidney disease, stroke, neuropathy)? (Check one)

| - 0-20% | - 21-40% | - 41-60% | - 61-80% | - 81-100% |
| --- | --- | --- | --- | --- |

**By individualizing, we mean choosing an A1C goal for each patient based on their characteristics**

1. Are you familiar with the concept of individualizing hemoglobin A1C (A1C) goals?

By individualizing, we mean choosing an A1C goal for each patient based on their characteristics (Check one)

- Yes
- No

1. To what extent, do you agree with individualizing A1C goals? (Check one)

- Strongly disagree
- Somewhat disagree
- Neither agree nor disagree
- Somewhat agree
- Strongly agree

1. How frequently do you individualize A1C goals? (Check one)
   - Never
   - Rarely
   - Sometimes
   - Most of the time
   - Always
2. In general, if your patient with type 2 diabetes has a stable A1C level for 1 year, do you ever initiate conversations about discontinuing or reducing the dose of their diabetes medications? (Check one)

- Yes
- No

1. At what A1C level do you initiate this conversation? (Check one)
   - A1C < 5.0%
   - A1C < 5.7%
   - A1C < 6.0%
   - A1C < 6.5%
   - A1C < 7.0%
   - A1C < 8.0%
   - The A1C level depends on the patient’s characteristics
   - Other: ____________________________________________________
2. In what other clinical situations do you initiate this conversation? (Check all that apply)
   - - - When a patient has symptoms that could be from their medications
       - When a patient is at risk for polypharmacy
       - When a patient may not be taking the medicine as prescribed
       - When a patient is diagnosed with a condition that significantly reduces their life expectancy
       - When a patient raises concerns about the costs of medications
       - Other: ____________________________________________________
3. What is your gender?

- Female
- Male

1. What year did you finish your highest level of clinical training? __________________________
2. Since training, how many years have you been in practice? __________________________
